# Comprehensive prognostic report of the Japanese Breast Cancer Society registry in 2006

**DOI:** 10.1007/s12282-015-0646-3

**Published:** 2015-10-13

**Authors:** Takayuki Iwamoto, Naohito Fukui, Takayuki Kinoshita, Keisei Anan, Naoki Niikura, Masaaki Kawai, Naoki Hayashi, Kouichiro Tsugawa, Kenjiro Aogi, Takanori Ishida, Hideji Masuoka, Shinobu Masuda, Kotaro Iijima, Seigo Nakamura, Yutaka Tokuda

**Affiliations:** Department of Breast and Endocrine Surgery, Okayama University Hospital, Okayama, Japan; The Japan Clinical Research Support Unit and the Public Health Research Foundation, Tokyo, Japan; Department of Breast Surgery, National Cancer Center Hospital, Tokyo, Japan; Department of Surgery, Kitakyushu Municipal Medical Center, Kitakyushu, Japan; Department of Breast and Endocrine Surgery, Tokai University School of Medicine, 143 Shimokasuya, Isehara, Kanagawa 259-1193 Japan; Department of Breast Surgery, Miyagi Cancer Center, Natori, Japan; Department of Breast Surgery, St. Luke’s International Hospital, Tokyo, Japan; Division of Breast and Endocrine Surgery, Department of Surgery, St. Marianna University School of Medicine, Kawasaki, Japan; Department of Breast Surgery, Shikoku Cancer Center, Matsuyama, Japan; Department of Surgical Oncology, Graduate School of Medicine, Tohoku University, Sendai, Japan; Sapporo-kotoni Breast Clinic, Sapporo, Japan; Department of Pathology, Nihon University School of Medicine, Tokyo, Japan; Department of Breast Oncology, Cancer Institute Hospital, Tokyo, Japan; Division of Breast Surgical Oncology, Department of Surgery, Showa University, Tokyo, Japan

**Keywords:** Breast cancer, Prognosis, Report, Japan, Registry, 2006, The Japanese Breast Cancer Society

## Preface

**Fig. 1:**
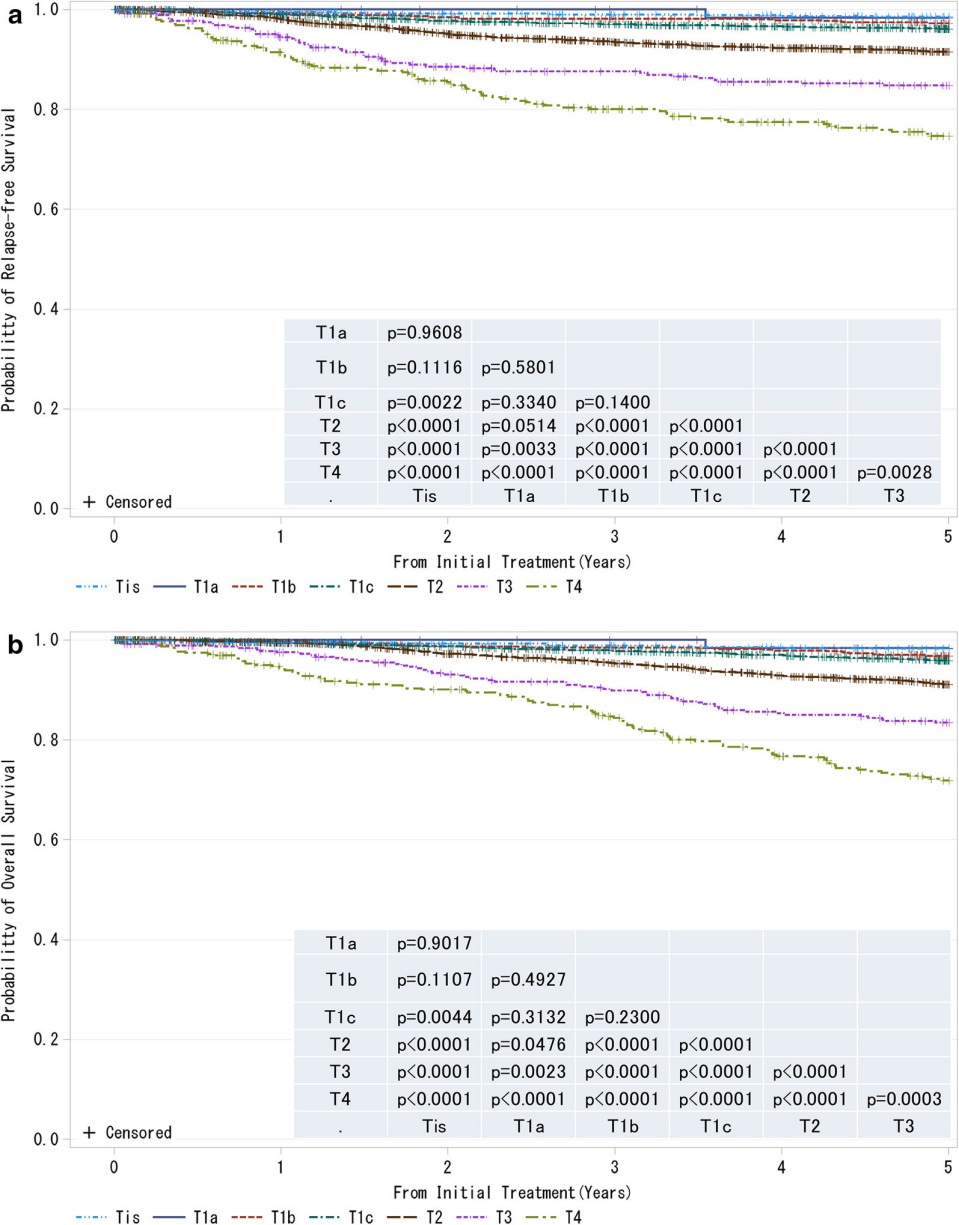
**a**, **b** Kaplan–Meier curves for relapse-free and overall survival of all cases by tumor classification (cT-category). *P* values were calculated using the log rank test. *Tis* non-invasive ductal carcinoma, lobular carcinoma in situ, or Paget disease, *T1a* ≤0.5 cm, *T1b* 0.5 < tumor ≤ 1.0 cm, *T1c* 1.0 < tumor ≤ 2.0 cm, *T2* 2.0 < tumor ≤ 5.0 cm, *T3* >5.0 cm, *T4* tumor of any size with direct extension to the chest wall and/or skin (ulceration or skin nodules) or inflammatory carcinoma

**Fig. 2:**
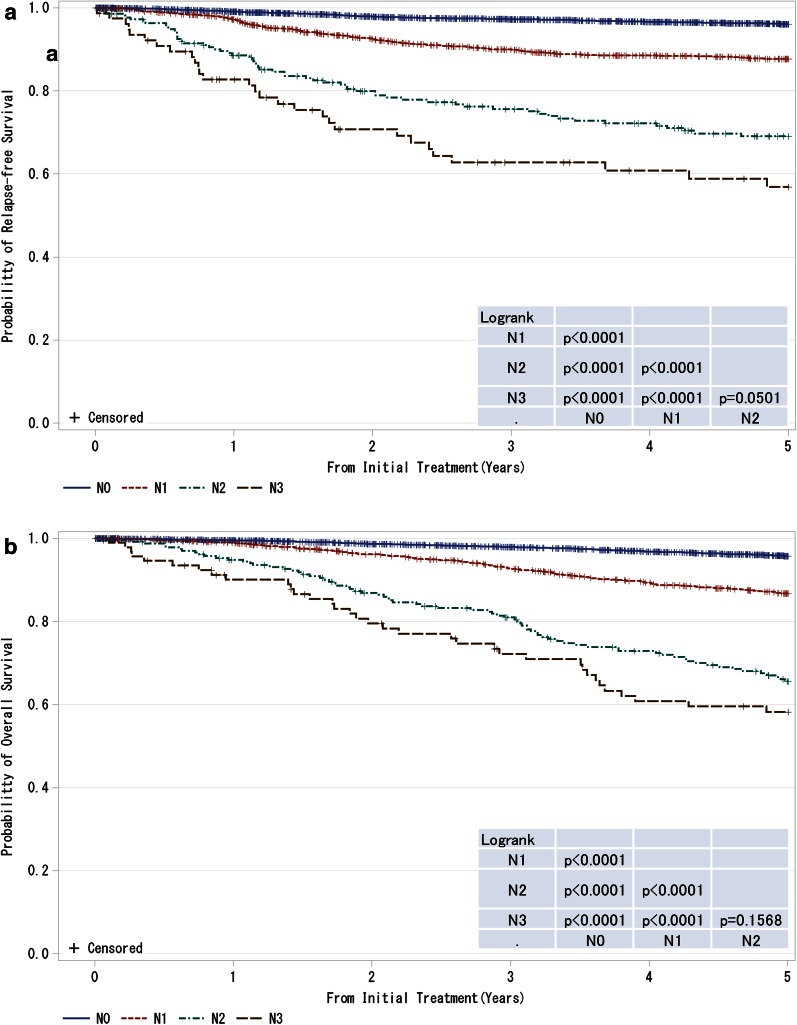
**a**, **b** Kaplan–Meier curves for relapse-free and overall survival of all cases by regional lymph nodes status (cN-category) *N0* no regional lymph node metastases, *N1* metastases in movable ipsilateral level I, II axillary lymph node(s), *N2* metastases in ipsilateral level I, II axillary lymph nodes that are clinically fixed or matted OR Metastases in clinically detected ipsilateral internal mammary nodes in the absence of clinically evident axillary lymph node metastases, *N3* metastases in ipsilateral infraclavicular (level III axillary) lymph node(s) with or without level I, II axillary lymph node involvement OR Metastases in clinically detected ipsilateral internal mammary lymph node(s) with clinically evident level I, II axillary lymph node metastases OR Metastases in ipsilateral supraclavicular lymph node(s) with or without axillary or internal mammary lymph node involvement. *P* values were calculated using the log rank test

**Fig. 3:**
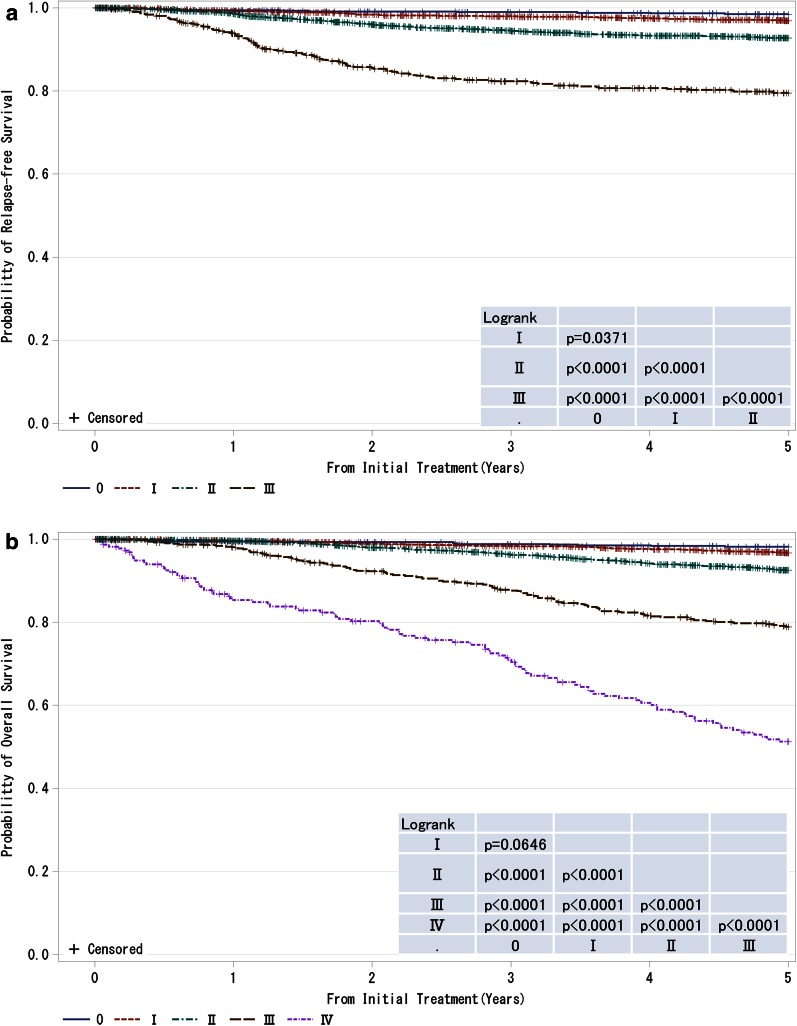
**a**, **b** Kaplan–Meier curves for relapse-free and overall survival of all cases by clinical stage (UICC). *P* values were calculated using the log rank test

**Fig. 4:**
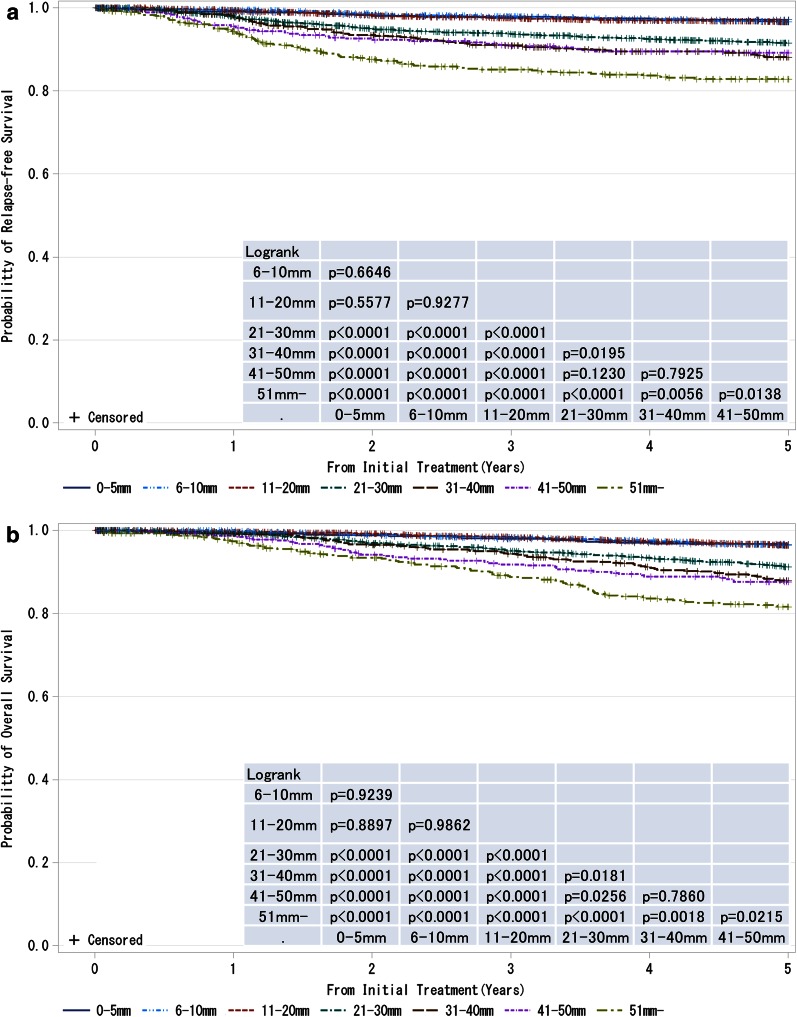
**a**, **b** Kaplan–Meier curves for relapse-free and overall survival of cases without neoadjuvant therapy by pathological tumor size (pT size). Tumor size is a marker of invasiveness. *P* values were calculated using the log rank test

**Fig. 5:**
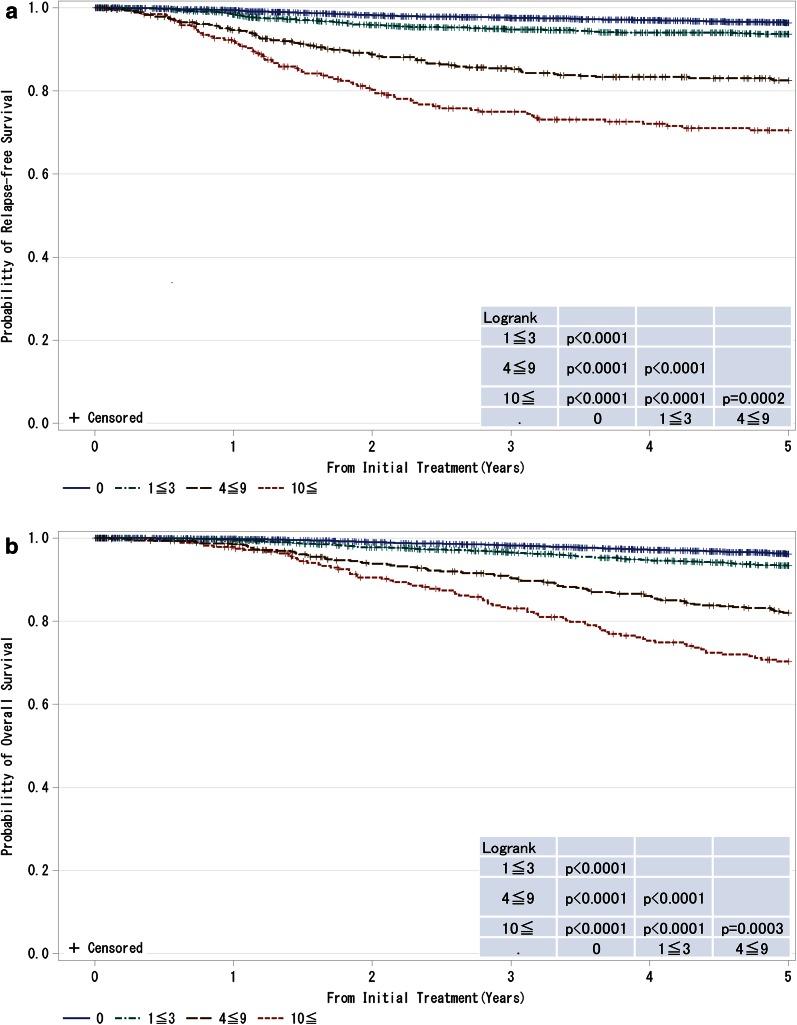
**a**, **b** Kaplan–Meier curves for relapse-free and overall survival of cases without neoadjuvant therapy by the number of metastatic lymph nodes. *P* values were calculated using the log rank test

**Fig. 6:**
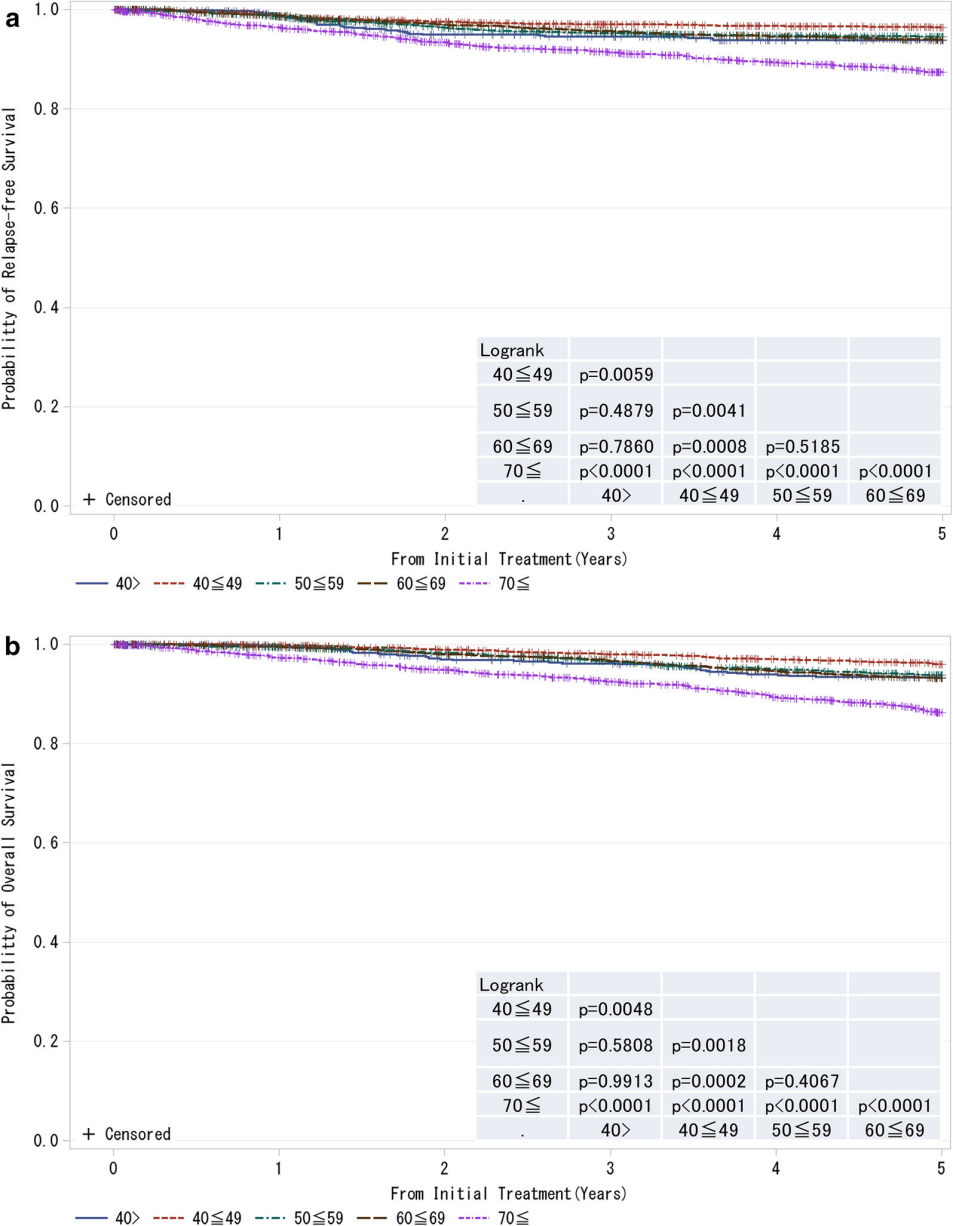
**a**, **b** Kaplan–Meier curves for relapse-free and overall survival of all cases by age. *P* values were calculated using the log rank test

**Fig. 7:**
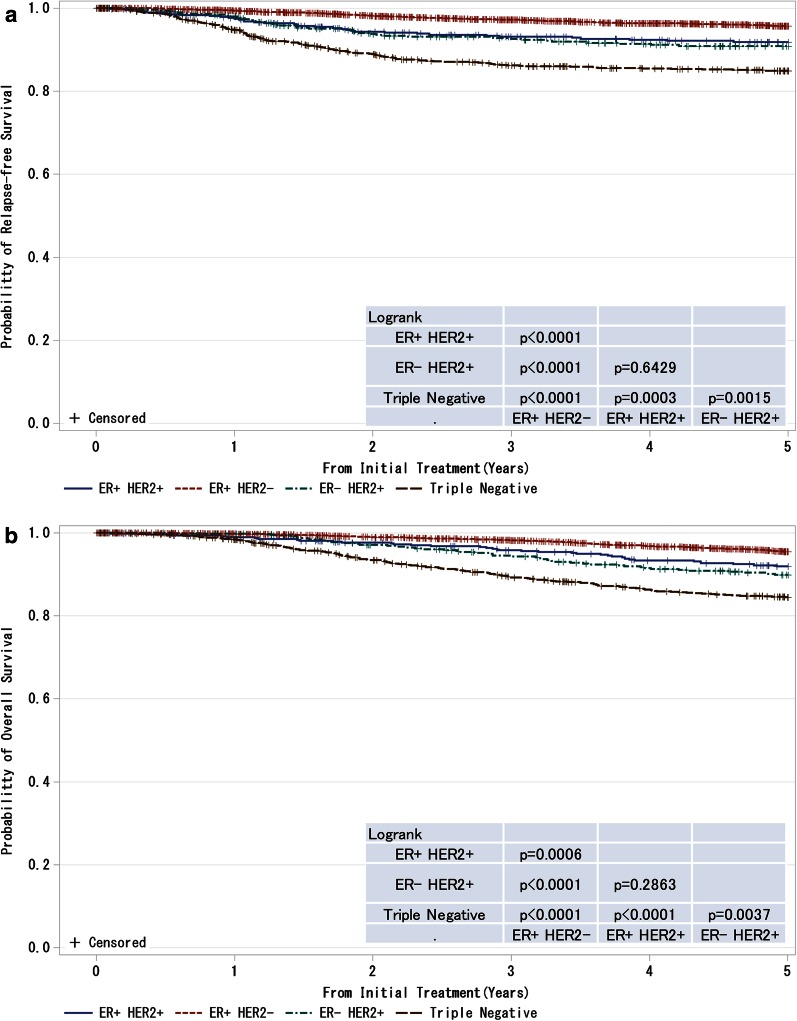
**a**, **b** Kaplan–Meier curves for relapse-free and overall survival of T1–T4, any N and M0 cases with respect to estrogen receptor (ER) status and HER2 (human epidermal growth factor receptor 2) amplification status. *P* values were calculated using the log rank test. Relapse-free survival and overall survival of patients with respect to combined ER and HER2 status

**Fig. 8:**
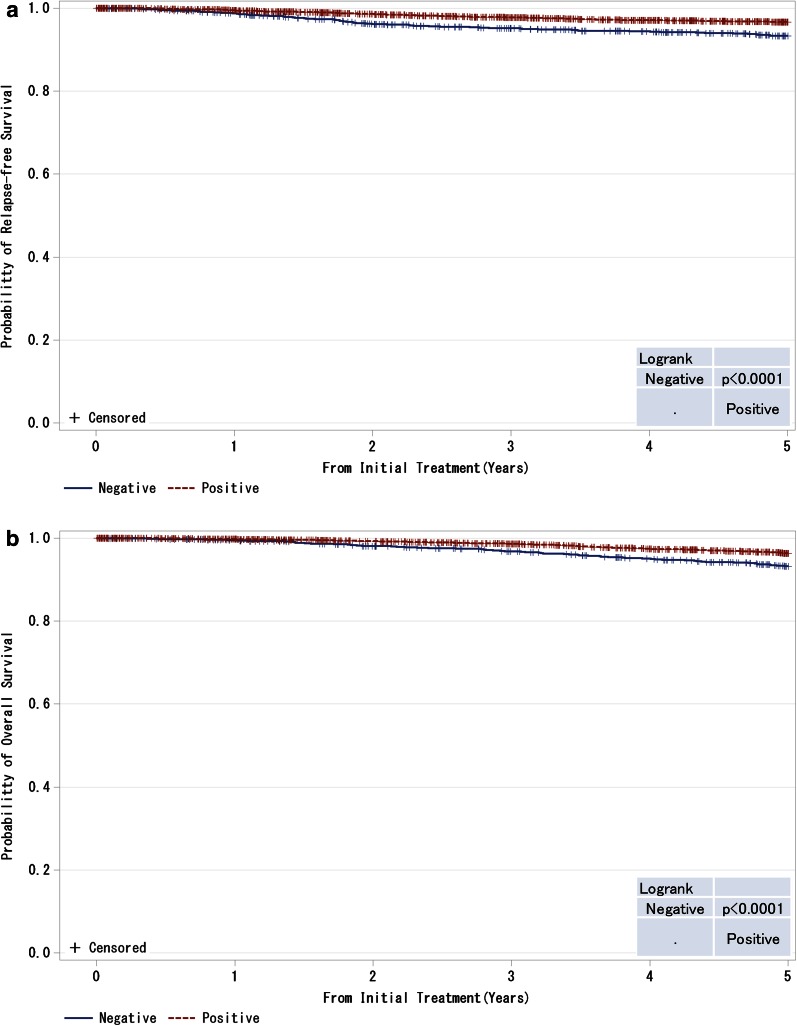
**a**, **b** Kaplan–Meier curves for relapse-free and overall survival of ER-positive and M0 cases by progesterone receptor (PgR) status. *P* values were calculated using the log rank test

**Fig. 9:**
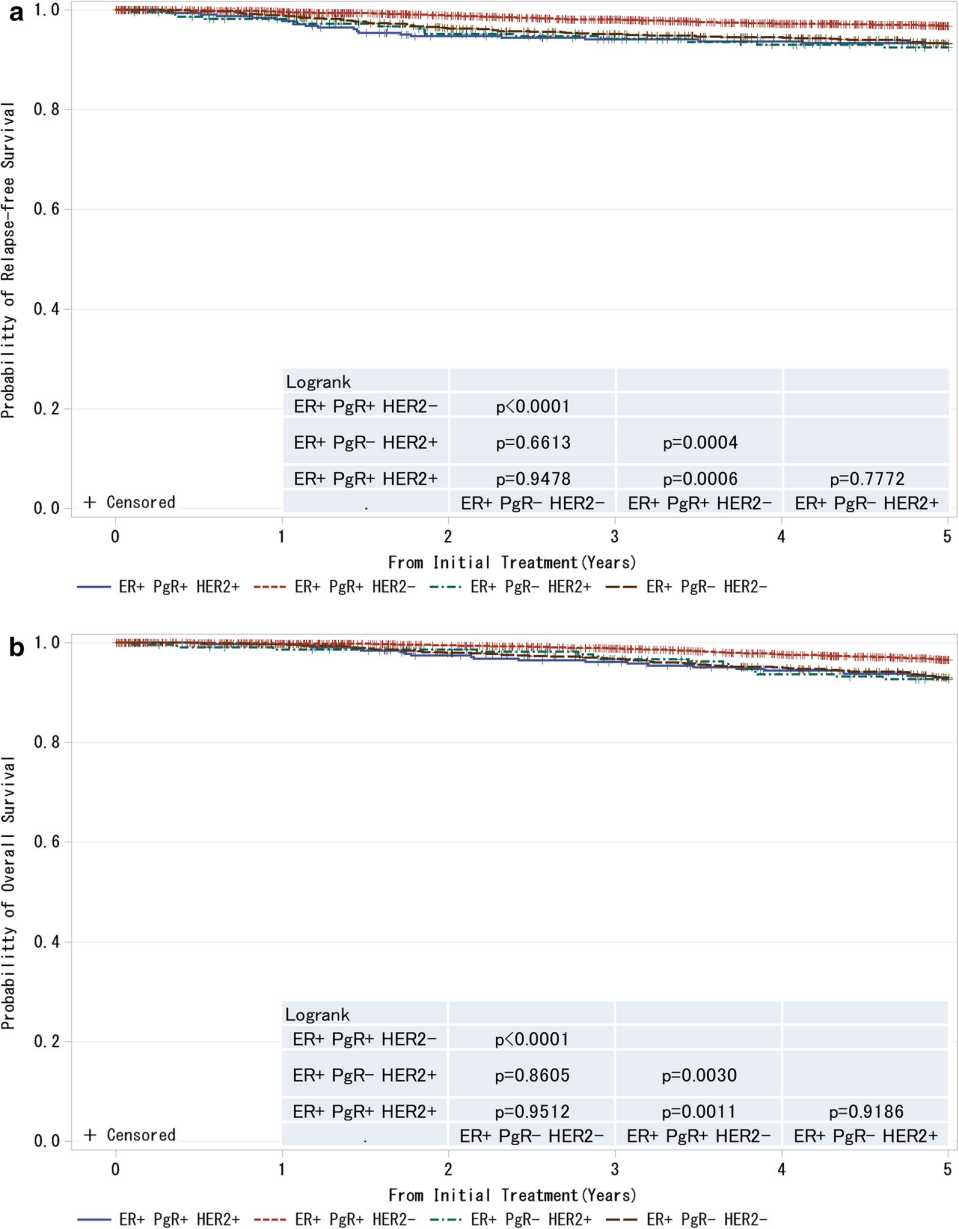
**a**, **b** Kaplan–Meier curves for relapse-free and overall survival of ER-positive and M0 cases with respect to PgR and HER2 amplifications. *P* values were calculated using the log rank test

The prognostic study for the Japanese Breast Cancer Society (JBCS) registry in 2006 was finally published here (Figs. 1, 2, 3, 4, 5, 6, 7, 8, 9, Sup. Table 1–9). The JBCS registry has been started from 1975. To 2003, for 29 years, 188,265 cases have been registered. With the cooperation of the Non-Profit Organization Japan Clinical Research Support Unit (J-CRSU) and the Public Health Research Foundation, we have moved to the new system by the web registration from 2004.

In 2006, the number of the registry for institutions was 352 and cases were 22,005. The number of institutions in this prognostic study was 134 and cases were 8788, with 39.9 %. An assessment of 5-year prognosis for cases registered in 2006 has been carried out, and here we report the results thanks to a number of efforts and cooperation. We believe that it is necessary to further promote the registry for contributions to improving breast cancer care and prognosis.

Background characteristics of the patients are summarized in Table 1. The 5-year disease-free survival (DFS) was 93.5 %, and the 5-year overall survival (OS) was 92.7 % at a median follow-up of 60.0 months (range 0.0–60.0). The TNM classification and histological classification were registered according to the UICC staging [1] and WHO classification systems [2], respectively. The present report includes age- and subtype-based analyses in addition to the traditional TNM classification-based analyses.

**Table 1:** Patient characteristics

Age		
Mean	S.D.	
57.42	12.94	
Tumor size(cm)		
Mean	S.D.	
2.60	2.05	
Tumor size	Count	%
T0	95	1.08
Tis	729	8.3
T1a	68	0.77
T1b	742	8.44
T1c	2599	29.57
T2	3043	34.63
T3	369	4.2
T4	390	4.44
Unknown	753	8.57
N		
N0	6758	76.90
N1	1614	18.37
N2	236	2.69
N3	92	1.05
Unknown	88	1.00
M		
M0	8464	96.31
M1	217	2.47
Unknown	107	1.22
Stage		
0	700	7.97
I	3010	34.25
II	3336	37.96
III	622	7.08
IV	217	2.47
Unknown	903	10.28
ER		
Positive	6514	74.12
Negative	2077	23.63
Unknown	197	2.24
PgR		
Positive	5168	58.81
Negative	3404	38.73
Unknown	216	2.46
HER2		
Positive	1230	14.00
Negative	6759	76.91
Unknown	799	9.09

The TNM classification was identified by the UICC staging system

*ER* estrogen receptor, *ER* estrogen receptor, *PgR* progesterone receptor, *HER2* human epidermal growth factor receptor 2

In addition to TNM classifications, estrogen receptor (ER), progesterone receptor (PgR), and human epidermal growth factor receptor 2 (HER2) statuses, which are strong prognostic factors, have become frequently used to determine the therapeutic strategy in the clinical setting. Note that during the study period, not only the cutoff level of ER, PgR and HER2 positivity, but also test procedures for immunostaining and HER2 gene amplification have not yet been standardized, and trastuzumab had been gradually spread in daily clinic in Japan. For ER-negative/HER2-positive patients, DFS improved from 85.0 % in 2004 to 90.9 % in 2006, and OS from 85.02 to 89.88 %, respectively.

We appreciate the considerable support that we have received and would like to ask for continuing understanding and support of the registry.

## Electronic supplementary material

Supplementary material 1 (DOCX 35 kb)
