# Supplementary material for: Comprehensive prognostic report of the Japanese Breast Cancer Society registry in 2006
Source: Breast Cancer. 2015 Oct 13;23:62–72. doi: 10.1007/s12282-015-0646-3 (PMC4691585; doi:10.1007/s12282-015-0646-3)
Supplement: Supplementary file 1 — Supplementary material 1 (DOCX 35 kb) [file 12282_2015_646_MOESM1_ESM.docx]

| Supplementary Table 1 Survival rate of all cases by tumor classification (cT-category) | | | | | | |
| --- | --- | --- | --- | --- | --- | --- |
| a. Relapse-free survival rate (%) | | | | | | |
| T | Number of Patients | 1 Year | 2 Year | 3 Year | 4 Year | 5 Year |
| Tis | 725 | 99.44 | 99.14 | 98.99 | 98.83 | 98.51 |
| T1a | 67 | 100.00 | 100.00 | 100.00 | 98.33 | 98.33 |
| T1b | 737 | 99.17 | 98.46 | 98.17 | 97.87 | 97.25 |
| T1c | 2,573 | 99.02 | 97.71 | 97.04 | 96.56 | 96.07 |
| T2 | 3,008 | 98.07 | 95.08 | 93.42 | 92.24 | 91.54 |
| T3 | 354 | 94.44 | 88.56 | 87.57 | 85.50 | 84.78 |
| T4 | 350 | 91.40 | 85.39 | 80.03 | 77.44 | 74.60 |
|  |  |  |  |  |  |  |
| b. Overall survival rate (%) | | | | | | |
| T | Number of Patients | 1 Year | 2 Year | 3 Year | 4 Year | 5 Year |
| Tis | 729 | 99.44 | 99.29 | 98.84 | 98.53 | 98.22 |
| T1a | 67 | 100.00 | 100.00 | 100.00 | 98.36 | 98.36 |
| T1b | 742 | 99.73 | 98.75 | 98.46 | 97.88 | 96.83 |
| T1c | 2,593 | 99.42 | 98.65 | 97.80 | 96.95 | 95.83 |
| T2 | 3,040 | 99.47 | 97.23 | 95.26 | 92.81 | 91.14 |
| T3 | 367 | 97.48 | 93.10 | 89.83 | 85.30 | 83.46 |
| T4 | 389 | 94.42 | 90.02 | 84.36 | 76.76 | 71.86 |

| Supplementary Table 2 Survival rate of all cases by regional lymph nodes status (cN-category) | | | | | | |
| --- | --- | --- | --- | --- | --- | --- |
| a. Relapse-free survival rate (%) | | | | | | |
| N | Number of Patients | 1 Year | 2 Year | 3 Year | 4 Year | 5 Year |
| N0 | 6,703 | 99.07 | 97.88 | 97.21 | 96.60 | 96.09 |
| N1 | 1,564 | 97.19 | 92.46 | 89.92 | 88.47 | 87.65 |
| N2 | 214 | 88.55 | 79.95 | 75.61 | 72.17 | 69.07 |
| N3 | 77 | 82.67 | 70.73 | 62.69 | 60.79 | 56.80 |
|  |  |  |  |  |  |  |
| b. Overall survival rate (%) | | | | | | |
| N | Number of Patients | 1 Year | 2 Year | 3 Year | 4 Year | 5 Year |
| N0 | 6,746 | 99.53 | 98.70 | 97.89 | 96.79 | 95.79 |
| N1 | 1,612 | 98.99 | 96.13 | 92.72 | 89.36 | 86.75 |
| N2 | 236 | 94.85 | 86.87 | 80.91 | 72.87 | 65.52 |
| N3 | 92 | 90.07 | 79.48 | 72.15 | 60.76 | 58.20 |

| Supplementary Table 3 Survival rate of all cases by clinical stage (UICC) | | | | | | |
| --- | --- | --- | --- | --- | --- | --- |
| a. Relapse-free survival rate (%) | | | | | | |
| Clinical stage | Number of Patients | 1 Year | 2 Year | 3 Year | 4 Year | 5 Year |
| 0 | 696 | 99.42 | 99.11 | 98.95 | 98.79 | 98.46 |
| Ⅰ | 2,988 | 99.22 | 98.34 | 97.88 | 97.47 | 96.93 |
| Ⅱ | 3,310 | 98.55 | 95.96 | 94.45 | 93.30 | 92.71 |
| Ⅲ | 610 | 93.74 | 85.49 | 82.25 | 80.64 | 79.54 |
|  |  |  |  |  |  |  |
| b. Overall survival rate (%) | | | | | | |
| Clinical stage | Number of Patients | 1 Year | 2 Year | 3 Year | 4 Year | 5 Year |
| 0 | 700 | 99.42 | 99.27 | 98.80 | 98.48 | 98.16 |
| Ⅰ | 3,004 | 99.60 | 98.91 | 98.31 | 97.58 | 96.73 |
| Ⅱ | 3,331 | 99.63 | 97.94 | 96.20 | 94.13 | 92.50 |
| Ⅲ | 620 | 98.01 | 92.18 | 87.62 | 81.53 | 78.93 |
| Ⅳ | 217 | 85.25 | 80.26 | 70.88 | 60.59 | 51.24 |

| Supplementary Table 4 Survival rate of cases without neoadjuvant therapy by pathological tumor size (pT size) | | | | | | |
| --- | --- | --- | --- | --- | --- | --- |
| a. Relapse-free survival rate (%) | | | | | | |
| pT size | Number of Patients | 1 Year | 2 Year | 3 Year | 4 Year | 5 Year |
| 0-5mm | 802 | 99.11 | 98.05 | 97.78 | 97.36 | 97.08 |
| 6-10mm | 958 | 99.47 | 98.60 | 97.93 | 97.11 | 96.62 |
| 11-20mm | 2,991 | 99.36 | 98.24 | 97.48 | 96.92 | 96.60 |
| 21-30mm | 1,709 | 97.71 | 94.88 | 93.63 | 92.47 | 91.46 |
| 31-40mm | 672 | 97.70 | 93.38 | 90.87 | 89.46 | 88.14 |
| 41-50mm | 354 | 95.43 | 92.21 | 90.68 | 89.41 | 89.08 |
| 51mm- | 510 | 94.32 | 87.62 | 85.08 | 83.61 | 82.83 |
|  |  |  |  |  |  |  |
| b. Overall survival rate (%) | | | | | | |
| pT size | Number of Patients | 1 Year | 2 Year | 3 Year | 4 Year | 5 Year |
| 0-5mm | 808 | 99.12 | 98.73 | 97.93 | 96.97 | 96.56 |
| 6-10mm | 964 | 100.00 | 98.93 | 98.27 | 97.47 | 96.42 |
| 11-20mm | 3,008 | 99.60 | 99.15 | 98.34 | 97.29 | 96.39 |
| 21-30mm | 1,719 | 99.35 | 96.94 | 94.99 | 93.37 | 91.25 |
| 31-40mm | 684 | 99.10 | 96.47 | 94.26 | 91.01 | 87.84 |
| 41-50mm | 364 | 98.61 | 94.07 | 91.75 | 88.81 | 87.60 |
| 51mm- | 526 | 97.26 | 93.38 | 88.75 | 83.57 | 81.56 |

| Supplementary Table 5 Survival rate of cases without neoadjuvant therapy by the number of metastatic lymph nodes | | | | | | |
| --- | --- | --- | --- | --- | --- | --- |
| a. Relapse-free survival rate (%) | | | | | | |
| n | Number of Patients | 1 Year | 2 Year | 3 Year | 4 Year | 5 Year |
| 0 | 5,148 | 99.39 | 98.18 | 97.53 | 96.99 | 96.41 |
| 1≦3 | 2,004 | 98.33 | 95.86 | 94.76 | 94.01 | 93.70 |
| 4≦9 | 512 | 94.63 | 88.73 | 85.40 | 83.30 | 82.53 |
| 10≦ | 267 | 91.88 | 80.26 | 74.91 | 72.05 | 70.47 |
|  |  |  |  |  |  |  |
| b. Overall survival rate (%) | | | | | | |
| n | Number of Patients | 1 Year | 2 Year | 3 Year | 4 Year | 5 Year |
| 0 | 5,175 | 99.74 | 98.98 | 98.13 | 97.15 | 96.20 |
| 1≦3 | 2,019 | 99.50 | 97.75 | 96.48 | 94.68 | 93.34 |
| 4≦9 | 523 | 98.44 | 93.80 | 90.50 | 86.08 | 81.99 |
| 10≦ | 277 | 97.81 | 90.52 | 82.99 | 75.30 | 70.33 |

| Supplementary Table 6 Survival rate of all cases by age | | | | | | |
| --- | --- | --- | --- | --- | --- | --- |
| a. Relapse-free survival rate (%) | | | | | | |
| Age | Number of Patients | 1 Year | 2 Year | 3 Year | 4 Year | 5 Year |
| 40> | 642 | 98.72 | 94.91 | 94.55 | 93.81 | 93.81 |
| 40≦49 | 1,904 | 98.56 | 97.52 | 96.95 | 96.71 | 96.41 |
| 50≦59 | 2,452 | 98.60 | 96.28 | 95.22 | 94.77 | 94.48 |
| 60≦69 | 1,949 | 98.80 | 96.91 | 95.64 | 94.56 | 93.91 |
| 70≦ | 1,620 | 96.35 | 93.39 | 91.32 | 89.33 | 87.35 |
|  |  |  |  |  |  |  |
| b. Overall survival rate (%) | | | | | | |
| Age | Number of Patients | 1 Year | 2 Year | 3 Year | 4 Year | 5 Year |
| 40> | 651 | 99.53 | 96.94 | 96.11 | 93.91 | 93.22 |
| 40≦49 | 1,926 | 99.84 | 98.87 | 97.93 | 96.98 | 95.94 |
| 50≦59 | 2,490 | 99.39 | 98.22 | 96.49 | 94.93 | 93.79 |
| 60≦69 | 1,986 | 99.44 | 97.97 | 96.63 | 94.62 | 93.12 |
| 70≦ | 1,646 | 97.29 | 94.81 | 92.41 | 89.31 | 86.23 |

| Supplementary Table 7 Survival rate of T1-T4, any N and M0 cases with respect to estrogen receptor (ER) status and HER2 (human EGFR-related 2) amplification status | | | | | | |
| --- | --- | --- | --- | --- | --- | --- |
| a. Relapse-free survival rate (%) | | | | | | |
| Breast cancer subtypes | Number of Patients | 1 Year | 2 Year | 3 Year | 4 Year | 5 Year |
| ER+ HER2- | 4672 | 99.39 | 98.13 | 97.20 | 96.30 | 95.64 |
| ER+ HER2+ | 470 | 97.41 | 94.26 | 93.07 | 92.32 | 91.80 |
| ER- HER2+ | 488 | 97.90 | 93.73 | 92.57 | 91.37 | 90.87 |
| Triple Negative | 1034 | 94.66 | 88.88 | 86.19 | 85.40 | 84.93 |
|  |  |  |  |  |  |  |
| b. Overall survival rate (%) | | | | | | |
| Breast cancer subtypes | Number of Patients | 1 Year | 2 Year | 3 Year | 4 Year | 5 Year |
| ER+ HER2- | 4696 | 99.78 | 98.96 | 98.21 | 96.77 | 95.47 |
| ER+ HER2+ | 472 | 98.93 | 97.63 | 95.84 | 93.32 | 91.93 |
| ER- HER2+ | 497 | 99.80 | 97.08 | 94.30 | 91.46 | 89.88 |
| Triple Negative | 1042 | 98.34 | 93.40 | 89.17 | 86.30 | 84.47 |

| Supplementary Table 8 Survival rate of ER-positive and M0 cases by progesterone receptor (PgR) status | | | | | | |
| --- | --- | --- | --- | --- | --- | --- |
| a. Relapse-free survival rate (%) | | | | | | |
| PgR | Number of Patients | 1 Year | 2 Year | 3 Year | 4 Year | 5 Year |
| Positive | 4,830 | 99.45 | 98.55 | 97.74 | 97.03 | 96.64 |
| Negative | 1,385 | 98.68 | 96.12 | 95.10 | 94.37 | 93.26 |
|  |  |  |  |  |  |  |
| b. Overall survival rate (%) | | | | | | |
| PgR | Number of Patients | 1 Year | 2 Year | 3 Year | 4 Year | 5 Year |
| Positive | 4,859 | 99.75 | 99.21 | 98.59 | 97.36 | 96.38 |
| Negative | 1,391 | 99.49 | 98.01 | 96.80 | 95.02 | 93.20 |

| Supplementary Table 9 Survival rate of ER-positive and M0 cases with respect to PgR and HER2 amplifications | | | | | | |
| --- | --- | --- | --- | --- | --- | --- |
| a. Relapse-free survival rate (%) | | | | | | |
| Breast cancer subtypes | Number of Patients | 1 Year | 2 Year | 3 Year | 4 Year | 5 Year |
| ER+ PgR- HER2- | 1,081 | 98.78 | 96.27 | 95.17 | 94.45 | 93.14 |
| ER+ PgR+ HER2- | 4,135 | 99.60 | 98.86 | 98.02 | 97.22 | 96.81 |
| ER+ PgR- HER2+ | 217 | 98.14 | 95.19 | 94.12 | 93.01 | 92.43 |
| ER+ PgR+ HER2+ | 310 | 97.71 | 94.72 | 94.03 | 93.67 | 93.30 |
| b. Overall survival rate (%) | | | | | | |
| Breast cancer subtypes | Number of Patients | 1 Year | 2 Year | 3 Year | 4 Year | 5 Year |
| ER+ PgR- HER2- | 1,086 | 99.72 | 97.91 | 96.74 | 95.07 | 93.02 |
| ER+ PgR+ HER2- | 4,158 | 99.83 | 99.42 | 98.80 | 97.58 | 96.56 |
| ER+ PgR- HER2+ | 218 | 98.62 | 98.62 | 96.67 | 93.68 | 92.68 |
| ER+ PgR+ HER2+ | 313 | 99.68 | 97.40 | 96.07 | 94.38 | 92.99 |
